# Enhancing Behavioral Health Competencies for Senior Center Staff: Lessons Learned From Workforce Training Efforts

**DOI:** 10.1093/geroni/igaa057.001

**Published:** 2020-12-16

**Authors:** Bronwyn Keefe, Jennifer Tripken

**Affiliations:** 1 Boston University, Boston, Massachusetts, United States; 2 National Council on Aging, Arlington, Virginia, United States

## Abstract

Increases in the numbers of older adults with mental health and substance use concerns compel us to identify best practices in training to address these issues. Senior Centers are an ideal location for behavioral health education programs as they are the go-to place for many older adults. This session will describe a program funded by The Retirement Research Foundation and offered in collaboration with Center for Aging and Disability Education and Research at Boston University and NCOA to increase senior center staff knowledge and skills. Approximately 250 senior center staff in Illinois, Florida, and Wisconsin completed an online certificate in Behavioral Health and Aging. Results show that 100% of respondents felt that the training was useful for their job; 93% felt that they will be a more effective worker as a result of the training; and 97% felt that the information they learned in the training will make a difference with the people they serve. We held key informant interviews to assess the impact of training and participants stated that their knowledge, skills, and behaviors were influenced by the program. At the organizational level, leaders reported new programming related to behavioral health and revised practices and protocols. This presentation will cover: (1) the extent to which training participants mastered the competencies needed for effective practice; (2) knowledge and skills gained from the training program; (3) Senior Centers’ capacity to identify and refer older adults to mental health services; and (4) organizational changes related to behavioral health programming with older adults.

